# Secondary Structure of the Novel Myosin Binding Domain WYR and Implications within Myosin Structure

**DOI:** 10.3390/biology10070603

**Published:** 2021-06-29

**Authors:** Lynda M. Menard, Neil B. Wood, Jim O. Vigoreaux

**Affiliations:** Department of Biology, University of Vermont, Burlington, VT 05405, USA; lynda.menard@uvm.edu (L.M.M.); neil.wood@uvm.edu (N.B.W.)

**Keywords:** myosin, flightin, flight muscle, coiled-coil, circular dichroism

## Abstract

**Simple Summary:**

WYR is a conserved protein domain characteristic of the muscle protein flightin, estimated to have originated in the ancestor to hexapods and crustaceans ~500 MYA. This study characterizes the secondary structure of WYR and shows that it binds the coiled-coil motif of muscle myosin and changes its structural properties. Characterizing WYR and its role in conjunction with myosin provides valuable insight into enduring evolutionary processes driving the success of Insecta. The relationship between WYR and myosin further reveals a means of ultrastructural regulation capable of informing the molecular structure–function relationships essential for the mechanical properties and structural stability of muscle.

**Abstract:**

Structural changes in the myosin II light meromyosin (LMM) that influence thick filament mechanical properties and muscle function are modulated by LMM-binding proteins. Flightin is an LMM-binding protein indispensable for the function of Drosophila indirect flight muscle (IFM). Flightin has a three-domain structure that includes WYR, a novel 52 aa domain conserved throughout Pancrustacea. In this study, we (i) test the hypothesis that WYR binds the LMM, (ii) characterize the secondary structure of WYR, and (iii) examine the structural impact WYR has on the LMM. Circular dichroism at 260–190 nm reveals a structural profile for WYR and supports an interaction between WYR and LMM. A WYR–LMM interaction is supported by co-sedimentation with a stoichiometry of ~2.4:1. The WYR–LMM interaction results in an overall increased coiled-coil content, while curtailing ɑ helical content. WYR is found to be composed of 15% turns, 31% antiparallel β, and 48% ‘other’ content. We propose a structural model of WYR consisting of an antiparallel β hairpin between Q92-K114 centered on an ASX or β turn around N102, with a G1 bulge at G117. The Drosophila LMM segment used, V1346-I1941, encompassing conserved skip residues 2-4, is found to possess a traditional helical profile but is interpreted as having <30% helical content by multiple methods of deconvolution. This low helicity may be affiliated with the dynamic behavior of the structure in solution or the inclusion of a known non-helical region in the C-terminus. Our results support the hypothesis that WYR binds the LMM and that this interaction brings about structural changes in the coiled-coil. These studies implicate flightin, via the WYR domain, for distinct shifts in LMM secondary structure that could influence the structural properties and stabilization of the thick filament, scaling to modulation of whole muscle function.

## 1. Introduction

The insect indirect flight muscle (IFM) is highly ordered, stretch activated and known to produce wing beats up to 1000 times per second [1,2]. The IFM of *Drosophila melanogaster* has been a valuable informant to structure–function relationships of myofibrillar proteins, mechanical parameters modulated for stretch activation, and as a model for investigating the molecular underpins of muscle and cardiac diseases [3,4,5,6,7]. Drosophila IFM has also served for the discovery and characterization of novel contractile proteins necessary to tune the structural and viscoelastic properties to optimize function. A well-studied example is flightin (UniProtKB—P35554), a 20 kDa protein shown to be a myosin-binding component of the thick filament by genetic, biochemical, and structural studies [8,9,10,11]. While *D. melanogaster* flightin is exclusive to the IFM, its wide-ranging presence in hexapods and crustaceans (Pancrustacea, sensu stricto) and its deep evolutionary history suggest a broader role in muscles, and perhaps in other tissues [10]. A comparative sequence analysis of flightin revealed a tripartite organization characterized by an ~52 amino acid conserved domain from H84 to T136 - denoted as WYR- that dates to the origin of Pancrustacea, bordered at the N-terminal and C-terminal sides by less conserved regions of variable length [10].

The analyses of flightin mutants in *D. melanogaster* have revealed its important role in flight and courtship, two behaviors that underscore the evolutionary success and prolific speciation of insects [7,12,13]. Specifically, mutants that express a truncated flightin missing the C-terminal region (*fln^ΔC44^*) are incapable of generating a courtship song or a wing beat to propel flight, similar to the flightin null mutant, (*fln^0^*). In contrast, mutants that express a truncated flightin missing the N-terminal region (*fln^ΔN62^*) have impaired flight mechanics and produce an abnormal courtship song that lessens the male’s mating success [12]. Despite the truncations, the mutant flightin variants remain integral components of the fiber indicating that neither the N-terminal nor C-terminal region is necessary for flightin incorporation into the thick filament. In this study, we test the hypothesis that the conserved WYR domain harbors a myosin rod binding sequence.

Flightin has been shown to impact the stability, structure and organization of IFM thick filaments, sarcomeres, myofibrils, and fibers. Fibers in mutants lacking flightin (*fln^0^*) bunch up upon eclosion [9] and fray and break when exposed to rigor regardless to prior exposure to mechanical activation [14]. Sarcomeres of the *fln^0^* pupal IFM are 25–30% longer than normal and there are fewer thick filaments across the diameter of myofibrils (17–19 vs. 25–26) [9]. The C-terminal mutant (*fln^ΔC44^*) exhibits slightly shorter (~9%) sarcomeres with dispersed or absent M-lines and a myofilament lattice that is less ordered and more compact (i.e., reduced inter-thick filament spacing) compared to a transgenic null rescued control line [15,16]. The N-terminal mutant (*fln^ΔN62^*) also exhibits slightly shorter sarcomeres that lack an evident H-zone and have a narrower M-line. Myofibrils contain more thick filaments and a more compact and less “crystalline” (regular) myofilament lattice [12,17]. 

IFM fibers that either lack flightin (*fln^0^*) or express a mutant form (*fln^ΔN62^*) and (*fln^ΔC44^*) manifest a variety of mechanical defects that include alterations in cross-bridge cycling kinetics, viscous and elastic moduli, and power output [12,14,16]. Native IFM thick filaments isolated from these mutant strains more clearly delineate the complex role that flightin plays in dictating thick filament structure, integrity and mechanical properties [17,18]. While the absence of flightin results in thick filaments that are substantially longer (and more fragile) than normal, truncation of the N-terminal region has no effect on thick filament length while truncation of the C-terminal region results in shorter thick filaments. The absence of flightin also results in a significant decrease in filament stiffness (persistence length) with the N-terminal region making a larger contribution to stiffness than the C-terminal region. 

Decreased accumulation of flightin is found in mutants of the myosin rod *Mhc^6^*, R1559H and *Mhc^13^*, E1554K [19] suggest that these residues, or influential intramolecular interactions in the local light meromyosin (LMM) coiled-coil, may be part of an LMM flightin binding site, as supported by in vitro studies [8].

Here, we use circular dichroism to test the hypothesis that WYR binds LMM (V1346 through I1941) and to characterize the secondary structural changes associated with WYR engagement. This study also represents the first experimental structural characterization for the novel domain, WYR. The WYR-LMM interaction is put into context by further analysis of the Lethocerus and Drosophila IFM thick filament cryo-EM model structures that include non-myosin densities along the length of the thick filament [20,21].

## 2. Materials and Methods

### 2.1. Purification of MHC Fragment

A 68.9 kDa 602 aa peptide encompassing V1346 through I1941 of D. melanogaster myosin heavy-chain (herein LMM) with an N-terminal 6× His-tag cloned into pET-23a vector was transformed into *E. coli* BL-21(DE3) pLysS cells [8]. Cells were grown in Luria broth (LB) containing 100 µg/mL ampicillin and 50 µg/mL chloramphenicol. Upon reaching A600 of 0.8–1.0, culture was chilled on ice for 15 min, and expression was induced by adding IPTG to 0.75 mM and incubating for 16 h at 25 °C with gentle rocking. Induced cells were collected by centrifugation at 10,000 rcf for 10 min and stored as pellets at −40 °C. Pellets were resuspended in lysis buffer described by Korkmaz et al. [22], and lysed by sonication. To purify the MHC peptide, the lysate was rocked at 4 °C with Ni-NTA-agarose resin for one hour in a 20 mL column before being rinsed with 8 column volumes of 20 mM imidazole wash-buffer. The resin was then rinsed in two 15 mL portions of each 40- and 80 mM imidazole wash buffer, with 10 min of rocking in each 15 mL portion. The peptide was eluted in six 1 mL fractions and one 12 mL fraction for a total volume of 18 mL 200 mM imidazole elution buffer. All wash and elution buffers are variants of ‘Buffer A’ described by Korkmaz et al. with the following modifications: imidazole concentration was adjusted to 20 mM, 40 mM or 80 mM for wash buffer and to 200 mM for elution buffer [22]. The relative purity of each fraction was assessed via SDS-PAGE stained with Krypton (Thermo Scientific, Rockford, IL, USA). Selected fractions were further purified and concentrated with an Amicon Ultra-15 50k centrifugal filter, and buffer exchanged into 400 mM NaF, 20 mM sodium phosphate pH 7.0, 1 mM TCEP for storage. Protein concentration was determined via absorbance at 280 nm using an extinction coefficient of 18450 cm^−1^ M^−1^. Densitometry using ImageJ was carried out on Krypton stained gels to ascertain purity of the sample [23] and the peptide identity verified by mass spectrometry.

### 2.2. WYR Peptide

Synthetic, 6.6 kDa 52 aa WYR peptide encompassing H84-T136 of D. melanogaster flightin was sourced from Genscript (Piscataway, NJ, USA). Peptide was suspended in ddH_2_O, filtered through a 0.22 µm Millipore filter, and concentration determined by micro BCA assay and absorbance at 280 nm using an extinction coefficient of 21,430 cm^−1^ M^−1^.

### 2.3. Circular Dichroism Sample Preparation

WYR and LMM samples ranging from 0.5 to 10 µM were prepared in 700 µL of 215 mM NaF, 20 mM Sodium Phosphate, pH 7, 1 mM TCEP. LMM preparations were centrifuged at 10,000 rcf for 10 min and filtered through a 0.22 µm Millipore filter. Blank samples used an equivalent volume of filtrate from the final flow through from LMM concentrators as used in the experimental samples to account for any possible trace contaminant. These blanks were checked against equivalent freshly prepared buffer. A rectangular STARNA quartz cuvette with a pathlength of 0.2 cm was used throughout. Between experiment days the cuvette received a full wash with the provided STARNA detergent in accordance to STARNA protocol [24]. Between samples, 1× 60% EtOH and 3× dH_2_O rinses were carried out and residual fluid was evaporated with pressurized nitrogen.

### 2.4. Circular Dichroism Measurements and Analysis

Samples were measured at 25 °C using a Jasco J-1700 spectropolarimeter at a scanning-speed of 20 nm/min with a digital integration time (DIT) of 8 s and bandwidth of 0.5–1 nm over six accumulations, minimally from 260–190 nm on continuous scan mode. A minimum *n* = 6 was used for measurements of the experimental combination of LMM and WYR and an *n* = 10 for WYR alone in 215 mM NaF, 20 mM Sodium Phosphate, pH 7, 1 mM TCEP. Wavelengths at maxima and minima, as well as θ_222/208_ and θ_192/208_ ellipticity ratios were recorded for all spectra. Structural interpretations were generated using BeStSel, after conversion to Mean Residue Ellipticity (MRE), for the range of 190–250 nm [25,26]. While other analysis programs exist, BeStSel is best suited for non-helical proteins of more unusual structure through both its diverse eight element modelling and inclusion of a basis dataset that incorporates proteins with rare secondary structures. BeStSel was most appropriate given the non-helical profile of WYR along with its high aromatic content and presence of proline.

Independent spectra for each peptide were evaluated after subtracting baseline spectra. To account for concentration and peptide molecular weight, the resultant ellipticity in millidegrees (mdeg) was converted to MRE using the following equation:

Ellipticity = m°*MRW/(10*L*C)(1)

where m° is the millidegrees ellipticity at a given wavelength, MRW is the mean residue weight which is molecular weight of the peptide divided by number of amino acids in length minus one (M/(N − 1)) in daltons, L is the cell pathlength in cm and C is the peptide concentration in g/L.

For experiments in which the two peptides were combined (LMMWYR), the experimental output (“Actual”) was compared to a value representing nonbinding conditions (“Theoretical”) attained by adding the independent ellipticities of LMM and WYR with buffer subtracted. Differences in magnitudes between “Actual” and the additive “Theoretical” CD profiles was indicative of binding.

To evaluate the structural profile of LMM and WYR together, the combined (LMMWYR) spectra with buffer subtracted were converted to molar ellipticity using two distinct methods: A Separated Parameters Method and a Combined Parameters Method.

### 2.5. Separated Parameters Method

For this method, we calculated the Theoretical (nonbinding) proportions of the spectra that WYR and LMM contribute at each wavelength ({λ∈ℤ|190 ≤ λ ≤ 260}) by dividing the total Theoretical mdeg output by WYR alone or LMM alone at each wavelength observed. This fraction was then used to separate the Actual mdeg output into two data sets, one representing the LMM proportion and the other representing the WYR proportion. The resultant set of values for the WYR proportion was then subjected to MRE conversion using WYR parameters (molecular weight, concentration) and the resultant set of values for the LMM proportion was subjected to MRE conversion using LMM parameters. These two sets of values were then added to attain Actual(separate) or “Act(sep)”. To avoid high error arising from differences in sign (±) between the LMM only and WYR only profiles used to estimate proportions, the baseline was shifted to evade sign change at the mdeg level and then re-established after MRE conversion.

### 2.6. Combined Parameters Method

This method treats LMM and WYR of the LMMWYR experimental output as one unit. In this case, the mdeg of LMMWYR is converted to MRE (Equation (1)) using combined parameters: the average molecular weight of LMM and WYR and the sum of their concentrations.

Further accommodation was made for differences in LMM concentration between experiments by equalizing the [LMM] for all experiments, also involved in the calculation of theoretical and actual outputs. To do this, the median mdeg value at the wavelength that exhibited the least variation between all LMM only outputs (215 nm) was used as a marker by which all LMM outputs were compared. The relative value attained, representing deviation in magnitude either above or below the median, was used to scale mdeg values for each experiment.

Additional measurements for helicity used equations based on the 222 nm magnitude [27], 208 nm magnitude [28] or 230–240 nm slope [29] and software-based predictions on larger ranges. In addition to BeStSel (the primary program used), K2D, CONTIN and CDSSTR programs from the Dichroweb suite [30,31] were used. CONTIN and CDSSTR output were based on the SMP180 reference dataset.

### 2.7. Co-sedimentation Assays

Co-sedimentation assays were performed with LMM titrated by WYR in quintuplicate. Components were added to a final buffer of 215 mM NaF, 20 mM Na-P, 0.5 mM TCEP in a total volume of 60 µL. Twenty microliters were removed and labelled Pre-Spin (PS). Solutions were incubated overnight at 4 °C and then centrifuged at 15,000 rcf at 4 °C for 10 min. Twenty microliters of supernatant (S) were separated without disturbing the pellet. The remaining 20 µL of solution were included in the pellet (P) fraction and accounted for in the calculations, as described in the next section. Sedimentation of LMM with Insulin (5.8 kDa, 51 aa) was used as a control, performed in triplicate, to determine non-specific binding/sedimentation. 

Samples were combined with 5 µL of 5× Sample Buffer (50% glycerol; 300 mM Tris HCl, pH 6.8; 10% SDS; 0.05% Bromo Blue, 125 mM DTT) and boiled for 10 min. The samples were loaded into 15 well 15% SDS PAGE minigels and run at 170 V. Gels were fixed in 40% Ethanol, 10% Acetic acid with one exchange at 20 min. They were rocked in this solution overnight. Krypton (Thermo Fisher) was used to stain the gels for 2.5 h and destained in accordance to Krypton protocol prior to viewing. Gels were viewed by Gel Doc (Bio-Rad) and the .tiff files were exported for analysis.

### 2.8. Densitometry

Image Studio Lite Ver 5.2 (LICOR) was used to assess band density of LMM and WYR. Intensity values from ‘S’ were subtracted from ‘P’ to attain adjusted P (P-S). To correct for non-binding precipitation, the ratio (P-S)/P was calculated for all trials and the (P-S)/P of LMM alone was subtracted for LMM intensity values in the presence of WYR; (P-S)/P of WYR alone was subtracted for WYR intensity values in the presence of LMM. Insulin (20 µM) was used as a negative control and any LMM that sedimented with Insulin was subtracted from the LMM sedimenting with WYR as non-specific binding. The fraction that pelleted beyond non-binding for both WYR and LMM was converted to µM quantities based on loading amount.

GraphPad PRISM was used to fit the specific binding and calculate parameters (Kd and Bmax). 

## 3. Results

### 3.1. WYR Structure Contains Antiparallel Beta Strands and Aperiodic Elements

**Figure 1:**
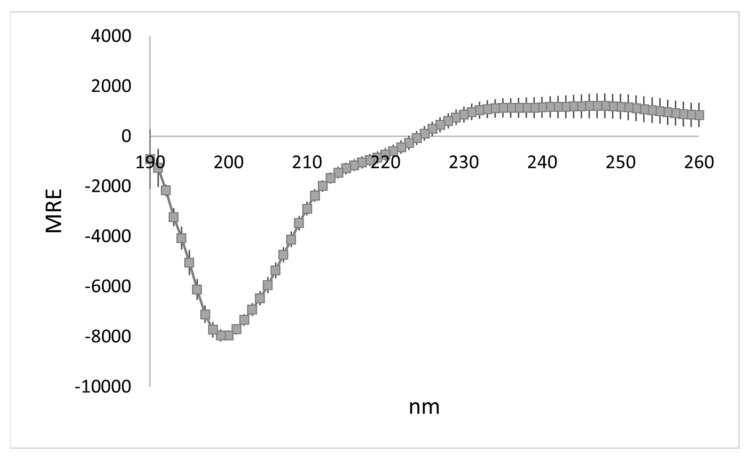
CD profile of WYR (10 µM) at 25 °C shows positive ellipticity >225 nm, a shoulder between 210–220 nm, a strong negative band at ~200 nm and a trough that remains in the negative at ~190 nm. N = 10. Each point represents mean with SEM.

The CD profile of WYR is characterized by a band at 190 nm, a negative band at 200 nm and a shoulder around 220 nm with the profile residing in the positive range >225 nm (Figure 1). It is distinctly nonhelical with a 222/208 of 0.38 ± 0.02. Deconvolution with BeStSel estimates WYR to be composed of ‘other’ (48%), antiparallel β (31%), and turn structures (15%) (Table 1). The CD profile is most reminiscent of right-twisted antiparallel β strands and unordered, irregular structure [26]. BeStSel breaks down the antiparallel β structure into left-twisted, relaxed and right-twisted. Seventy-four percent of the antiparallel β structure is in the right-twisted form (Table 1).

We then examined if differences in WYR concentration impact the secondary structure estimations. Increasing WYR concentration leads to changes in the spectral profile at wavelengths below 200 nm, decreasing the magnitude of the negative band and shifting it to 205 nm, and plateauing the 190 nm band to 200 nm. The shoulder at 220 nm is retained (Figure S1). No notable changes in secondary structure occurred when decreasing the concentration five-fold (to 2 µM). However, increasing the concentration four-fold (40 µM) showed large increases in helical content with concomitant decreases in antiparallel β content and, to a lesser degree, ‘other’ content (Figure S2). For a 52-aa peptide, at least 7.7% must be estimated helical for an α helix to be present, making the lower content estimations for all but the highest WYR concentration most likely due to non-helical contributions and possible multimerization.

In order to locate possible β turn structure, we used NetTurnP, a program that evaluates the proclivity of a sequence towards different types of β turns, based on individual amino acid propensities and would-be associations (e.g., i, i + 3) with nearby residues [32]. The prediction along the length of the WYR sequence is shown in Figure S3. The region between K114 and Q126 shows the highest probability of containing β turns. Type II, Via1 and Vib are considered the most probable within the region of greatest β turn likelihood. Type IV and Type I are consistently high between K114 and Q126. Though Type IV shares a similar pattern with the general β turn propensity pattern, Type IV is unlikely as an X-Pro cis amide bond is required between the i + 1 and i + 2 residues with X likely being an aromatic residue [33]; the prolines of WYR are not immediately neighboring the conserved aromatic residues.

We also tested programs that predict secondary structure from primary sequence. This was performed in order to see where β or turn content is predicted in the WYR sequence and whether there were regions of consensus that corroborate findings by CD. Secondary structure estimates for WYR were obtained with CFSSP [34], YASPIN [35], PHDpsi [36], PSIPRED [37], Phyre2 [38], Jpred [39,40], and i-tasser [41,42,43]. We examined the output of these along with all the secondary structure prediction methods offered by Network Protein Sequence Analysis (NPS@) of Pôle Bioinformatique Lyonnais [44], PredictProtein [45] and the Proteus Structure Prediction Server [46] amounting to a total of 22 programs (Figure S4 and Supplementary text). While it is known that peptides with high aromatic content like WYR result in a greater challenge for structure prediction [47], these programs are still useful in evaluating areas of potential consistency within the sequence. Algorithms that remain coherent in the context of experimental findings may be particularly informative.

Compared to the β sheet content obtained from the WYR CD profile, 21 of the 22 programs predicted substantially less or no β sheet/strand content with a much higher projection of helical content. YASPIN, PsiPred, Reprofsec (PredictProtein), Porter 4.0 and Jpred (Jnet) all predicted no β strand content along the entirety of the WYR sequence (Figure S5A) and ranged from a high of 71% (YASPIN) to a low of 40% (Jpred) helical content with the remainder being random coil (not shown). We changed all the tyrosines in the sequence to alanines, a change expected to favor β over α helical content in the context of the WYR sequence, to examine the extent to which the prediction was impacted for these five programs to generally evaluate the consideration of aromatic character. Types of change in the structure were very variable among the five programs (Figure S5B). Only one pair of programs (JPred and ReProfSec) were in consensus about a designation change of one residue (K94 becoming helical) and only one residue gained a β designation (Q92) in only one program (JPred). GOR I, SOPM from NPS@, and CFSSP detected turn regions at positions K89, R100-N102, K113-T116, R124-P125, and R134 (Figure S6). GOR I was the only program that detected bulk extended β strand/sheet content (Figure S7). These findings are unsurprising in the context of the unique WYR sequence, as structural predictions for sequences without homologs tend to be inaccurate [48].

### 3.2. WYR Binds the LMM

**Figure 2:**
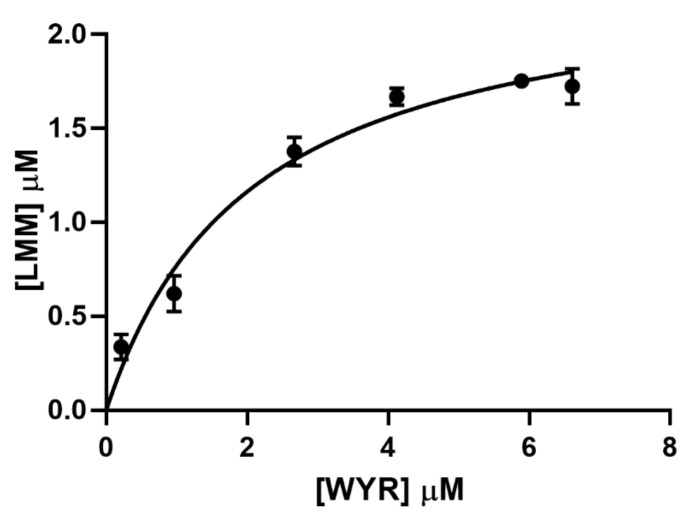
WYR promotes LMM sedimentation. Each point represents mean ± SEM; N = 3–5.

We assessed the interaction between WYR and a myosin peptide representing the C-terminal 600 residues of the myosin LMM using a co-sedimentation assay. The assay was carried out at WYR to LMM ratios ranging from 1:2 to 10:1 under conditions that do not favor LMM polymerization. As shown in Figure 2, sedimentation increases with increasing concentrations of WYR. From the amount of co-sedimenting WYR we obtained a Kd of 2.06 ± 0.29 µM (95% Cl: 1.548–2.762) and Bmax of 2.36 ± 0.11 µM (95% Cl: 2.157–2.625). Maximal desolvation was attained at a stoichiometry of WYR:LMM of 2.4:1.

**Figure 3:**
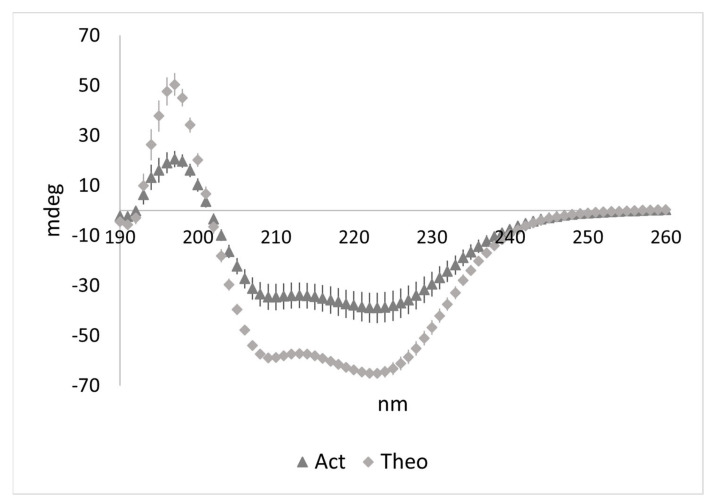
Actual experimental output of LMMWYR compared to the Theoretical output expected for a nonbinding LMM+WYR combination supports binding of LMM and WYR. N = 6. Each symbol represents mean ± SEM.

We also determined binding between the flightin and myosin peptides using circular dichroism. This was achieved by obtaining ‘Actual’ (Act) experimental mdeg output for a solution containing both (LMM+WYR) peptides and comparing it to the ‘Theoretical’ (Theo) mdeg output, the sum combination of the experimental values for solutions of LMM alone and WYR alone (Figure 3). The Act and Theo profiles would be the same under conditions of non-binding or if no structural change took place upon binding. As shown in Figure 3, the magnitudes of the Act and Theo profiles are notably different from each other, indicative of binding, while the pattern remained reminiscent of an α helix. The 222/208 ratios are significantly different (Actual: 1.13 ± 0.06; Theoretical: 1.05 ± 0.07; *p* < 0.05) at the mdeg level, and more qualitative information can be gleaned after MRE conversion.

The comparison of the Act and Theo mdeg output is also useful in determining the existence of structural change but information about each of the components involved in binding is limited. The signals for WYR and LMM are not separated out in the Act experimental output and the proportion of the signal due to each component is not evident. As WYR is smaller than the LMM segment by a factor of ~10 (6.6 kD vs. 68.9 kD), spectral profile from WYR alone experiences an approximate 10X magnitude adjustment relative to LMM spectra when these are converted to MRE. At the mdeg level, LMM spectra naturally dominates as evidenced by the persistence of the helical-like profile seen in Figure 3.

**Figure 4:**
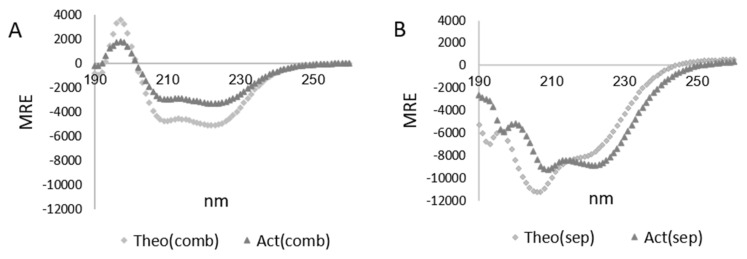
Comparison of MRE methods for experimentally combined WYR and LMM (Act) and projected non-binding profiles (Theo). (**A**) Comparison of Act and Theo using the Combined (comb) method for MRE calculation. (**B**) Comparison of Act and Theo using the Separate (sep) method for MRE calculation.

The non-equal elliptical shifts in magnitude due to a size difference between WYR and the LMM, along with their qualitatively different spectra (Figure S8), are a necessary consideration when it comes to conversion to MRE. As mdeg ellipticity at a given wavelength differs between the separate WYR and LMM CD spectra, each species’ elliptical contribution is expected to vary across the combined (LMM+WYR) spectrum. Under the condition of non-binding, the proportionality is determined using the Theo spectrum in which the ratios WYR/(LMM+WYR) and LMM/(LMM+WYR) are determined to attribute a non-binding proportion at each wavelength. When taking this into consideration, the mdeg values of Act can also be separated by non-binding proportions. Once the Act output is split, each set of values is converted to MRE and then added together. This is referred to here as Actual (separate) or Act(sep). Act(sep) can be directly compared to Theo MRE (separate LMM and WYR MRE values added together) referred here as Theo(sep) (Figure 4B). Since the contribution from WYR and LMM to the secondary structure of the bound form is likely to be different from that of the unbound form, applied in Act(sep), conversion of the experimental LMMWYR Act mdeg to MRE was also carried out, in which LMM and WYR were treated as a combined unit, incorporating weighted averaging of concentrations and MW, designated Act(combined) or Act(comb). Both Act(sep) and Act(comb) can be evaluated on their own and compared to an MRE converted theoretical output using the corresponding processing method (Figure 4). Act(sep) and Act(comb) demarcate the ends of a structural range of conserved proportionality to single unit, and analysis of both by BeStSel gives a more inclusive perspective on the secondary structure present in the bound state.

### 3.3. Characterization of the LMMWYR Structure

A helical-like profile dominates for both Theo(comb) and Act(comb) (Figure 4A). Theo(sep) and Act(sep) retain some elements of a helical-like profile at the ππ*∥ (208 nm) and nπ* (222 nm) transitions with Act(sep) exhibiting a small hypsochromic shift in the overall profile especially notable at, and below, 200 nm and in the area of sign change (240–250 nm). This qualitative change observed in Act(sep) could be due to an impactful hypsochromic shift of the ππ*⊥ component at 195 nm and a relative strengthening of the nπ* (222 nm) transition.

As sedimentation experiments suggested that ~30% of LMM sediment in the presence of WYR upon centrifugation at the ratio used in CD experiments, we examined whether sedimentation could account for the magnitude decrease observed. Due to the Act(comb) profile being similar to that of LMM alone, we were able to assess whether the signal intensity decrease between Act(comb) and LMM alone matched what would be expected from sedimentation alone. Act(comb) contains both WYR and LMM so this comparison posits that WYR complexes with LMM with minimal impact on existent structure. We found that, between 200–240 nm, the average signal intensity is 55% for Act(comb) compared to LMM alone with a median relative decrease to 67%. This suggests that 33–45% of the structural component would have to be removed (sedimented out) to account for the shift in magnitude. Given that maximally ~30% of LMM is found to sediment with WYR under centrifugal conditions, the decrease in magnitude is not strictly due to desolvation and there is an additional component shift.

Using a variety of methods for estimation of helical content [26,28,49,50,51,52,53], we obtained a range of helicities from 5–23.7% for Act(comb) and 14.1–45.6% for Act(sep) compared to 15.1–29.8% for Theo(comb) and 19.3–51.6% for Theo(sep), respectively (Table 2).

If we view output from Act(sep) to Act(comb) as a range of structural possibilities from conserved proportionality to single unit calculations (Table S1), BeStSel reports 7.4–16.9% helicity; 17.9–31% Antiparallel β, 0–1.2% Parallel β, 15.2–17.6% Turn, and 45.2–47.6% ‘Other’ content. Greatest variation is seen for helical and antiparallel β content. Both Act(sep) and Act(comb) exhibit decreased helicity compared to their Theo counterparts in BeStSel, as well as Contin and CDSSTR (Table 2).

The 222/208 ratios, indicative of coiled-coil nature at higher values, are higher in the Actual experimental combination of LMM and WYR compared to the Theoretical non-binding scenarios in both conserved proportionality ‘Sep’ and single unit ‘Comb’ methods. Using the combined method, the 222/208 ratio is found to be 1.1 for Theo(comb) and 1.2 for Act(comb). The 222/208 ratio is 0.7 for Theo(sep) and 1.0 for Act(sep).

### 3.4. Characterization of LMM Structure

Previous studies have shown Myosin, LMM, and segments of the LMM to exhibit traditional helical character in which a maximum is present at ~195 nm and two minima exist at 208 nm and 222 nm [27,28,54,55,56,57]. We obtained similar results over a five-fold range of LMM concentrations (2 µM to 10 µM) (Figure S9). A 222/208 ratio of 0.9–1 is indicative of α helical content and >1.1 is indicative of coiled-coil content. The observed 222/208 ratios are helical in nature at both low and high concentrations (1.08 for low; 1.17 for high) with the ratio being more indicative of coiled-coil content at the higher LMM concentration. However, we did not find the magnitudes at 208 nm or 222 nm intense enough to suggest bulk alpha helical content of more than 30% when using the typical methods of calculation (Table S2). The low helicity calculated, despite the prevalence of a predominantly helical profile, may be due to the absence of a trigger sequence (T1308-E1321) [27], and inclusion of a non-helical tail region (V1928-I1941) in the LMM construct used in this study, among other factors. 

Various methods and basis values have been used for evaluating expected helicity. Many studies have focused on the magnitude at 222 nm. Some studies have focused on points other than 222 nm along the spectrum within the 220–230 nm region [53], the slope between 230–240 nm [29] or at 208 nm [28]. To evaluate helicity in the LMM construct used in this study, we tested several of these methods and several CD processing programs based on various gating parameters (K2D, CONTIN, CDSSTR, BeStSel) (Table S2).

The helical estimates for the two LMM concentrations were similar, with estimates at the higher concentration ranging from 8.2–28.7% and at lower concentration ranging from 8–28.5% α helical content. The greatest difference between the two concentrations is observed for CDSSTR and BeStSel. If results from programs that utilize protein libraries are removed (CONTIN, CDSSTR, BeStSel), helicity estimations range from 15.3 to 28.7% for the high concentration and from 14.2 to 28.5% for the low concentration. Slightly improved helical contents for higher concentrations may be indicative of LMM-LMM association. 

## 4. Discussion

In this study, we present evidence for the binding of the flightin WYR domain to the C-terminal 600 residues of myosin LMM and have identified structural changes in the LMMWYR complex. The complex of LMM and WYR sediments upon centrifugation and exhibits decreased helicity and increased coiled-coil nature compared to non-binding theoretical cases. We present the first structural analysis of the evolutionarily conserved WYR region and propose a hypothetical WYR structure containing antiparallel β strands, turns, and aperiodic ‘Other’ content in light of both our findings by CD and extant literature dedicated to secondary structure identification and classification. The LMM–WYR relationship is examined further with consideration to the nature of helical coiled-coils and myosin behavior in the context of muscle.

### 4.1. WYR Structure

**Figure 5:**
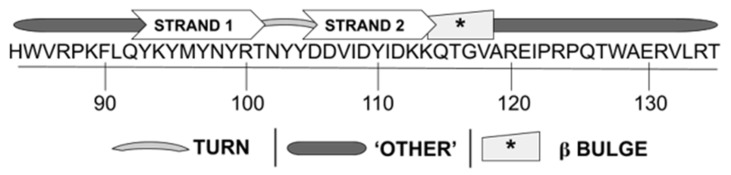
Pictograph model of WYR secondary structure elements. The numerical axis represents the #aa associated within the *D. melanogaster* flightin sequence. Strand 1 is the first strand of a 2-strand antiparallel β hairpin and is separated from Strand 2 by a turn segment proposed to exist between T101-Y104. C-terminal to Strand 2 is a β turn/G1 β bulge reliant on G117. Flanking the β and turn components is additional ‘Other’ content which may include loop and further turn structures.

The WYR structure, broken down into 48% ‘Other’, 31% antiparallel β sheet, and 15% turn content can be further elucidated by combining information from its CD profile with examination of the primary sequence to provide insight into specific regions most likely to be responsible for these structural segments. This analysis has yielded a model structure for WYR (Figure 5). In this structure, a β hairpin is present between Q92-K114, centered on a turn around N102 with a G1 bulge present around G117; N- and C-terminal to these structures contain ‘Other’ content likely to include loops and turns. Our reasoning towards this structure is as follows.

Model β sheets are generally characterized by a negative band at 218 nm and a positive band between 190–220 nm [58,59], with more highly twisted β sheets exhibiting more intense bands with opposite signs. Beta rich proteins have been further characterized into βI- and βII- types in which βII exhibit a poly(Pro)II-type (P_2_)-like profile [59] in which the positive band generally associated with typical β sheets (~198 nm) is countered by a negative band in the same vicinity (~200 nm) from the P_2_ component. P_2_ and βII profiles are frequently characterized as having high ‘other’ content as the P_2_ spectral profile is similar to those of non-periodic or denatured proteins. βII-types have a characteristic 200 nm negative band, some with a small positive band around 190 nm and a negative shoulder around 220 nm. While the WYR sequence contains several prolines, P_2_-type structure does not require prolines and is characterized by Ramachandran angles (φ, ψ) of approximately (−70°, +150°). βII structure is characterized as having the fraction of P_2_ content to β content equal to or greater than 0.4 [59]. The βII-type profile fits WYR well and ~30% β content would imply at least 12% P_2_ content (6–7 residues), though there may be more than 12% of the WYR structure adopting such a format.

Beta hairpins, the simplest antiparallel β sheets requiring only two β strands, are the most likely β content present in WYR. With the 52 aa WYR segment, 31% of residues potentially engaging in antiparallel β form would equate to the involvement of approximately 16 residues which could involve 2–3 β strands. Salt bridges often stabilize the surface of β sheets. The centrally located charged residues in WYR (K94, R100, D105, D106, D109) may be conducive to this structure. Aromatic residues are favored in the middle of β sheets while prolines are favored towards the edges and could be involved in the nucleation of β structure [60,61]. The alternating pattern of tyrosines spanning seven residues (Y93 to Y99) are supportive of an area central to a β strand which would permit a solvent-exposed tyrosine ladder on one side. The next likely strand is C-terminal to the first strand, closer to the central region of WYR, and requires separation from the first strand by a turn. Prolines in the WYR sequence (P88, P123, P125) are not permissive to β structure unless they are involved in its nucleation, so it is expected the entirety of β content lies between, and exclusive of, residues N-terminal to P88 and C-terminal to P123. 

Although the turn types possible are expansive, we suspect that a β type I’, II’, or ASX turn connects the two strands of the proposed WYR β hairpin. When considering the turn content, it is worth noting that turns can be nearly as variable as ‘other’ content and included in ‘other’ content, but tend to be defined with greater discretion. Beta turns are the most common type of nonrepetitive secondary structure [62] and are classically defined as possessing hydrogen bonding between the carbonyl of the residue at position i and NH of i + 2 or i + 3 with a distance of <7 Å between residues i and i + 3 [63]. These bonds are easy to form and break and are considered unstable unless further stabilized by side chain interactions [64], which may take the form of π–π, OH–π, or CH–π interactions with involvement of the tyrosine content. Type I’ and II’ are most conducive to right-twisted β strands [64,65], the predominant conformation indicated by CD/BeStSel. Type II and II’ structure specifically has preference for prolines and tyrosines as well as lysines in the i to i + 3 positions. As antiparallel β hairpin structures strongly prefer Type I’ or II’ turns, with these turns being not overly reliant on the presence of proline and glycine, this kind of β turn content is more likely to be present within the antiparallel β sheet of WYR. The region of TNYY (T101-Y104) is permissive of a β turn with T101 in the i position and N102 in the i + 1, or with N102 in the i position [66,67]. N102 is also conducive to tyrosine-asparagine stacking [68,69] while still permitting favorable interactions between the two tyrosines, presenting this as a possible site for an Asx turn. Asx turns fall under similar categorization as β turns but the side chain of residue i (Asn or Asp) hydrogen bonds to the backbone NH group of i + 2. Asparagine has been observed making such turn-based contacts with tyrosine at a position +2 residues away [70]; N102 and Y104 would be amenable to such interaction. Similarly, in ST turns, the serine or threonine at position i frequently will form a hydrogen bond with the main chain NH of i + 2. Given the expected 30°/aa rotation along the right twisted strand, T101 is in an ideal position to cap the end of the stacked OH/π bonds along the tyrosine ladder. However, if an ST turn were present, threonine’s R-group would not be available for engagement of the tyrosine ladder. A β type I’ turn or ASX turn would accommodate the expected twist well while leaving the threonine’s R-group to engage with tyrosine’s aryl group. Beta turns can also incorporate Asx turns and asparagine is strongly over-represented in β type I’ turns [71]; these are not mutually exclusive designations. Further support for N102 being more important to the structure than T101 is implicated in the very high conservation of the N102 residue and relatively poor conservation of the T101 residue among the WYR sequences characterized to date [10].

Programs that predict secondary structure from primary sequence alone are coherent with the proposed WYR structural model in a couple ways. Programs capable of predicting turns (GOR I, SOPM from NPS@, and CFSSP) have identified the region at and around N102 to be a probable turn as well as K114, the end of the proposed Strand 2 (Figure S6). All three of these programs likewise predict consistent, usually β, structure flanking the N102 turn region. When all programs are considered (Figure S4), the greatest proportion of β content is observed within Strand 1 with the highest coil/other content N-terminal to Strand 1 and N-terminal to K114, the proposed termination to Strand 2. 

‘Other’ content may also include some turn content as there can be overlapping turn segments in loops as in other turns, similar to β turns encompassing Asx turns. Pi-turns (*n* = 6; i, i + 5 H bonding) are most often present at the end of helical structures and often internalize a β-turn or are composed of multiple types of β-turns [72] as do over 30% of smaller α-turns [73]. Pi-turns are not likely for any part of WYR, partially due to the absence of helical content but also due to a general lack of glycines, strongly preferred in π-turns. Aromatics, of which there are many in WYR, are preferred at π-turn positions at i − 1, i + 6, but are also not appropriately positioned to other π-turn preferred residues that do exist within the WYR sequence. Loop structure on the outskirts of the central antiparallel β content (Q92-K114), however, could include γ-, β- or α-turn content.

Beta bulges, which may also be contributing to irregularity designated by ‘other’ content, occur in a sheet composed of at least two strands. Our proposed model includes a beta bulge adjacent to the antiparallel β hairpin, at G117. A type I turn followed by a G1 bulge is often a component of 3:5 hairpins and could be taking part in further brief antiparallel β content contact N-terminal to the conserved proline (P123). Of all the types of bulges, the G1 bulge is most often found on the outside ends of an antiparallel sheet, rather than being internal [74]. G1 bulges occur at the loop end of an antiparallel strand and are characterized by a glycine [75]. 

A notable characteristic of the WYR CD profile is the lack of distinct aromatic bands in the far UV (data not shown). Exciton coupling between the π–π* transitions in aromatics occur when they are in close proximity and this gives rise to distinct band patterns [76,77]. The aromatic contribution has been considered to be “idiosyncratic” among proteins [78] and could be masked by periodicity in the peptide backbone exhibiting dominant bands in an overlapping region. Both ionization of the aromatic groups and hydrogen bonding, along with other interactions can change the absorption profile for tyrosine and tryptophan [79,80,81], the most prevalent aromatic residues of WYR. The absence of far UV band patterns is suggestive that the aromatic residues of WYR are engaging in contacts that either ablate or substantially alter the presence and orientation of π–π* transitions. This is valuable information when considering the possible inter-residue contacts that influence WYR structure and drive its behavior in the presence of ionic solvents and binding partners.

Overall, the antiparallel β content is expected to be present as a β hairpin between Q92-K114 and the associated turn is expected to be a β type I’/ASX turn, possibly a β type II’ (Figure 5). C-terminal loop structures following a G1 β-bulge towards the end of the central hairpin, likely encompassing additional turns, are also proposed. Although we hypothesize the β hairpin of WYR to be centered on a type I’/II’ β turn or Asx turn, other portions of WYR are expected to exhibit additional turn or loop behavior given the BeStSel projected 15% turn and 48% other content. Such series-turn or loop structures may dominate in the C-terminal region of WYR and be involved with additional contacts with the central hairpin. 

A density associated with flightin has been identified within the thick filament of both Lethocerus [21] and Drosophila flight muscle [20] and is amenable to consideration alongside the proposed WYR structure. The density is nearly identical between the Lethocerus and Drosophila reconstructions and takes the form of a V-shape with a globular domain towards the hollow core of the thick filament. WYR is the only conserved region of flightin between these two organisms and would be expected to have the same secondary structure. In Drosophila, the ‘V’ region is expected to be ~20 residues in length, or ~38% of the whole density (N. Daneshparvar, personal communication, 27 January 2021). This would encompass H84-Y103 of WYR if oriented N to C terminal towards the center, or T135-T116 if in the reverse orientation. Both orientations terminate at, or very close to, highly conserved residues (Y103 or G117) with Y103 being implicated in the beta turn and G117 implicated in the G1 bulge. While it is not possible to know the actual orientation, either orientation would support the position where the shift to globular from the V-shape is made as involved in an acute structural transition, well aligned with the behavior of a highly conserved turn or bulge. 

### 4.2. LMMWYR Structure

The actual LMMWYR CD profile was shown to be distinct from the theoretical non-binding LMM+WYR profile and accompanied by a statistically significant difference between the 222/208 ratios of the base data (Actual: 1.13 ± 0.06; Theoretical: 1.05 ± 0.07; *p*-value < 0.05), which took on a helical-like profile in either case due to the dominance of the LMM. The 222/208 ratio is known to indicate the extent of coiled-coil nature, with 1.1 and higher indicating more coiled-coil behavior and a value closer to 0.9 for alpha helices less involved in coiled-coil behavior. The 222/208 ratio of the experimental combination of these peptides indicates that the LMMWYR complex features increased coiled-coil behavior that we attribute to the LMM as WYR alone features no helical content. Further processing of the LMMWYR CD spectra supports increased coiled-coil content in that both methods of processing into MRE of the Actual data result in higher 222/208 ratios than the corresponding Theoretical values (222/208 of 1.0 vs. 0.7 for Separate; 222/208 of 1.2 vs. 1.1 for Combined).

While the coiled-coil content is increased in the LMMWYR profile compared to the LMM profile, the helical content, determined by seven computational methods (BeStSel, CONTIN, CDSSTR, K2D, and equations based on 222 nm, 208 nm magnitudes or the 230–240 nm slope) is commonly shown to decrease. Act(sep) has 16.9–45.6% predicted helicity while Theo(sep) is found to be 19.3–51.6%. Act(comb) ranged from 5–23.7%, decreased compared to Theo(comb) ranging 16.5–29.8%. In both cases, the lower and higher ends are decreased in Actual compared to the corresponding Theoretical. This suggests decreased helicity despite the increased 222/208 ratio that indicate a coiled-coil presence. The decrease largely or exclusively originates from the LMM as WYR is not amenable to helical content.

Increased coiled-coil behavior coordinated with decreased helicity is explained upon consideration of the helical patterning that results in coiled-coil formation. The coiled-coil character of the LMM is driven by a heptad repeat, a character of primary structure, in which amino acids of each participating α helix in a dimer are arranged in a ‘HPPHCPC’ pattern, with H representing hydrophobic residues, P polar residues and C charged residues. The heptad is described as being in positions ‘abcdefg’ (Figure S10). The charged ‘e’ and ‘g’ positions of each helix stabilize the seam of the coiled-coil/super helix but can be responsible for instability in a single α helix. Hydrophobic interactions at the ‘a’ and ‘d’ positions form an apolar core that may engage in other structures when not in a coiled-coil form. However, the myosin heptad repeat contains many discontinuities: stutters, stammers and four conserved skip regions. Deviation from the heptad pattern is described as a ‘stutter’ if there is a deletion of 3 residues from the repeat, a ‘stammer’ for a deletion of 4 residues or a ‘skip’ for a deletion of 6 residues (or an extra 1 residue). With the presence of stutters, stammers, and skips, the coiled-coil is inconsistent along the length of the protein which can present as changes in pitch due to overwinding or underwinding (as from stutters and stammers) or ablation of the coiled-coil, as from skips [82]. Of the four conserved skip residues in the LMM (Drosophila Myosin [P05661]: T1187, E1384, E1581, G1806), the 2nd, 3rd and 4th are present in the peptide used in this study (V1346-I1941 segment). 

Accommodation of heptad irregularities may increase the overall presence of coiled-coils. As areas of unwinding in the coiled-coil structure exist due to imperfect heptad repeats, there remains more open helical structure such that there are helices not engaged in formation of a coiled-coil. Skips of the LMM inhibit the ability of helices N- and C-terminal to the discontinuity to form coiled coils; the skip residues responsible for unwinding of the coiled-coil result in a distortion that extends over 4 heptads in length in the LMM [83]. Stabilization within areas of heptad disturbance may permit the re-structuring necessary to prevent that disruption from being transmitted as extensively. Binding of WYR to the LMM may behave as a clasp, engaged in such re-structuring, giving rise to conditions promoting coiled-coil formation for the flanking helices. 

The LMM skips 3 and 4 feature conserved, exposed hydrophobic residues [83] that may play a role in binding interactions with myosin-affiliated thick filament proteins, such as flightin, and result in decreases in helicity despite increased coiled-coil content. Open individual helices tend to be less stable and more exposed than coiled-coils, naturally more accessible to ligands. The binding of such open-regions of the LMM would likely result in decreased helical content within those regions as the residues normally responsible for maintaining the H-bonding pattern and charge interactions are re-ordered or re-purposed for contacts with WYR. Hence, while the coiled-coil forming ability for heptads flanking heptad-deviated helices may be increased by WYR binding, the helices normally formed with imperfect heptad structure may form a different secondary structure after such binding. This would result in coordinate decreases in overall helical content along with the increases in coiled-coil formation, as observed in our findings.

The LMM’s coiled-coil structure and associated binding proteins work together to drive myosin assembly into higher order structures and incorporation into thick filaments. Some of this assembly is inherent to the LMM, driven by an assembly competence domain (ACD) and an alternating charge repeat pattern that spans 28 residues. In low ionic strength solutions (~150 mM), the LMM from rabbit striated muscle forms ordered aggregates (paracrystals) in vitro with a 43 nm periodicity similar to that found in vivo [84,85] though the assemblies vary in length and width. This behavior has also been identified in invertebrate and non-muscle myosin LMM [7,86,87] and has been employed in co-sedimentation binding studies [88]. Regular assembly according to species-specific filament parameters, however, require orchestration on the part of particular LMM-associating proteins, including molecular ‘rulers’ such as kettin and projectin, present in vivo. It’s known that flightin plays a role in thick filament length determination [9,17,18,89] though the mechanism is not fully elucidated. 

Sedimentation of the LMM in the presence of WYR supports a role for flightin’s involvement in the formation of higher order assemblies. As the sedimentation experiments were performed at a higher ionic strength (215 mM NaF) than that shown to result in paracrystal formation, WYR could be acting to permit such assembly of higher order structures at non-permissive ionic strengths or by a separate means, connecting LMM dimers together by binding different segments of the dimers simultaneously. As the in vitro stoichiometry for WYR:LMM is 2.4, there appear to be multiple binding sites which, if bound by different portions of the WYR peptide, may tie together multiple LMM dimers.

The WYR to myosin stoichiometry found in this study is comparable to the flightin to myosin stoichiometry found in previous studies. One study that used the same LMM fragment as in this study [8] found a flightin to myosin stoichiometry of 1:1 to 1:2 by solid state binding assays. Two other studies that examined the myosin structure within the thick filament of Lethocerus and Drosophila by cryo-EM [20,21] found the ratio of a non-myosin “red density” to myosin to be 1:1. Evidence points to this red density being a segment of flightin and most likely WYR [8,21,90]. The LMM segment used in this study contains three of the interacting sites observed for the ‘red density’. Thus, the 2.4:1 WYR:LMM we observed in vitro falls within the 1:1 ratio observed in the cryo-EM structures and the 3:1 ratio expected if WYR binds monomeric myosin. It is worth noting that our results with isolated LMM and WYR may not precisely reflect their in vivo relationship as there may be sites on the LMM that are accessible in vitro but inaccessible in vivo, or vice versa, due to association with other proteins. 

### 4.3. Implications for Thick Filament Structure and Mechanics

Flightin is a potential guide to thick filament quaternary structure through its multiple interacting sites along the length of the LMM and positioning to connect myosin dimers [20,21,90]. Its impact on thick filament and sarcomere length may be driven by a role for WYR in promoting ordered myosin assembly. Though the myosin coiled-coils can form assemblies on their own, more strict and species-specific alignment brought on by myosin-binding proteins is necessary to tune thick filament length and the arrangement of myosin dimers within it. This would explain the coincidence in the timing of flightin expression and the period of thick filament and sarcomere lengthening during Drosophila flight muscle development [91,92]. 

In addition to the organization of higher-order structure, myosin dimers being secured to each other at multiple points along the coiled-coil’s length would impact the mechanical characteristics of the thick filament. Variations in filament compliance are likely contributors to the differences in force production and force transmission across muscle types [93]. Insect flight muscle is characterized by high resting stiffness and a pronounced and delayed increase in tension after stretch, features that underpin its high power output capability. A mechanical ordering by partitioning the myosin LMM backbone into segments within which stiffness could be separately modulated would impact force production of myosin motors differently along the length of filament, allowing for a more regional and compartmentalized control of myosin motor behavior. The association of myosin dimers enables dispersal of forces and strategic organization of this assembly may be necessary to the flight muscle system for force production, force transduction, resilience against fracture, high power output, and efficacy of the system as a whole. Additional insight into the function of flightin could be gained from studies of non-homologous proteins with analogous functional properties. A notable candidate is myosin binding protein C, a protein shown to influence thick filament length and flexural rigidity in a manner analogous to flightin [89,94]. Flightin [90] and myosin binding protein C [88] share a common binding site in the LMM and it remains to be determined if through this interaction they impart common structural-mechanistic effects.

## 5. Conclusions

In this study, we have demonstrated binding between the flightin WYR domain and the myosin LMM while characterizing the resultant LMMWYR structure and the WYR structure. We propose the WYR structure is a βII type with the antiparallel β content hypothesized to be in the form of a β hairpin inclusive of the alternating tyrosines Y93-Y99, with the highly conserved N102-Y104 participating in the turn central to the hairpin. We propose the decreased α helical content and increased coiled-coil nature exhibited by LMMWYR results from the binding interaction occurring in areas of the LMM that are already unwound, though still participating in α helical structure [90]. This structural observation coupled with the co-sedimentation output supporting the development of higher order structures may allude to flightin’s role, via WYR, in thick filament assembly. Thick filament structure from cryo-EM studies and the known mechanical deficits in flightin mutants further support the organization of such assembly being relevant to structural integrity. How the relationship between flightin and the LMM impacts the mechanical capacity of the thick filament, stretch activation, and overall operation of the IFM merits further investigation.

## Figures and Tables

**Table 1:** BeStSel Secondary structure estimations for WYR.

2° Structure		% of Total
Other		47.9 ± 1.0
Antiparallel β	Right-twisted	23.2 ± 1.6
	Relaxed	7.8 ± 1.5
	Left-twisted	0.4 ± 0.2
Turn		14.7 ± 0.3
Helix	Alpha	0.8 ± 0.4
	Distorted	3.5 ± 1.1
Parallel β		1.7 ± 0.8

**Table 2:** Helical Prediction for LMMWYR by Various Methods.

Method	Act(sep)	Theo(sep)	Act(comb)	Theo(comb)
208 nm magnitude	45.6%	51.6%	23.7%	29.8%
222 nm magnitude	24.5%	21.5%	16.2%	15.1%
230–240 nm slope	24.3%	19.3%	10.1%	15.4%
K2D	30%	21%	10%	23%
Contin	14.1%	19.7%	9.9%	17%
CDSSTR	22%	24%	5%	16.6%
BeStSel (190–250 nm)	16.9%	21.4%	7.4%	16.5%

## Data Availability

The data presented in this study are available in the article entitled “Secondary structure of the novel myosin binding domain WYR and implications within myosin structure” and the accompanying supplement, and here https://scholarworks.uvm.edu/graddis/1341/ accessed on 22 June 2021.

## Supplementary Materials

The following are available online at https://www.mdpi.com/article/10.3390/biology10070603/s1, Figure S1: WYR in dH2O at concentrations from 2 µM to 40 µM shows bathochromic shifts at the higher concentrations for the near-positive at ~190 nm and minima at ~200 nm, Figure S2: WYR achieves higher helical content at increasing concentrations, as estimated by BeStSel (190-250 nm), Figure S3: NetTurnP prediction of beta turn propensity along the WYR sequence. The greatest propensity is predicted between K114 and Q126. The dark blue “Beta Turn” line represents probability of any type of beta turn, Figure S4: Secondary structure designation of the WYR sequence for Helical (H), Extended beta strand (E), and Random Coil (C). Shown is the consensus of 22 programs (see preceding text for listing) in which a greater number of identified instances reflects greater consensus, Figure S5: (A) Secondary structure designation for WYR structure from 5 programs capable of finding Extended beta strand content (E) but only detecting Helical (H) or Random Coil (C) content. (B) Secondary structure designations for WYR from the same 5 programs with all tyrosines have been changed to alanines- shown on the x-axis with stars, Figure S6: Secondary structure designation from three turn-inclusive programs finding Helical (H), Extended beta (E), Turn (T), and Coil (C) content, Figure S7: Secondary structure designation by GOR1. ‘e’ is for extended beta strand, ‘h’ is for helical, ‘t’ is for turn and ‘c’ is for random coil identification, Figure S8: WYR-alone (light grey squares) and LMM-alone (dark grey circles) Mean Residue Ellipticity (MRE) at 10 µM, Figure S9: MRE CD profile of LMM displays a characteristic helical profile at 10 µM and 2 µM from 190-260 nm. The 193-194 nm maxima observed at 2 µM shows a bathochromic shift to 197-198 nm at the higher concentration, Figure S10: Two alpha helices form a coiled-coil via their ’heptad’ repeats in which positions ’a’ and ’d’ form a hydrophobic core and charged residues at positions ’e’ and ’g’ add further stabilization. Although HPPHCPC is the ideal heptad pattern, HXXHXXX is also an accepted heptad pattern. H=hydrophobic, P=polar, C=charged, X can be any type of residue, Table S1: BeStSel structural predictions for Act(sep), Act(comb), Theo(sep) and Theo(comb) for 190-250 nm, Table S2: Fractional alpha helical composition for 10 µM and 2 µM LMM spectral profiles according to various methods of calculation,
