# Supplementary material for: Secondary Structure of the Novel Myosin Binding Domain WYR and Implications within Myosin Structure
Source: Biology (Basel). 2021 Jun 29;10(7):603. doi: 10.3390/biology10070603 (PMC8301185; doi:10.3390/biology10070603)
Supplement: Supplementary file 1 [file biology-10-00603-s001.zip › biology-1260816-supplementary.pdf]

Supplementary Materials to accompany the article "Secondary Structure of the Novel Myosin Binding Domain WYR and Implications within Myosin Structure"

Lynda Menard, Neil Wood, and Jim Vigoreaux

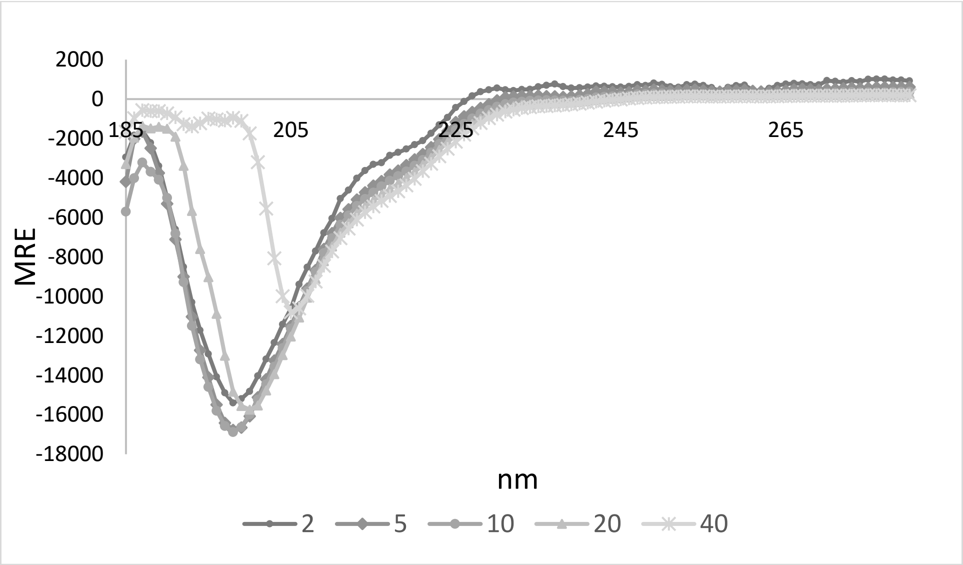

Supplementary Figure S1. WYR in dH2O at concentrations from 2 µM to 40 µM shows bathochromic shifts at the higher concentrations for the near-positive at ~190 nm and minima at ~200 nm). N=1.

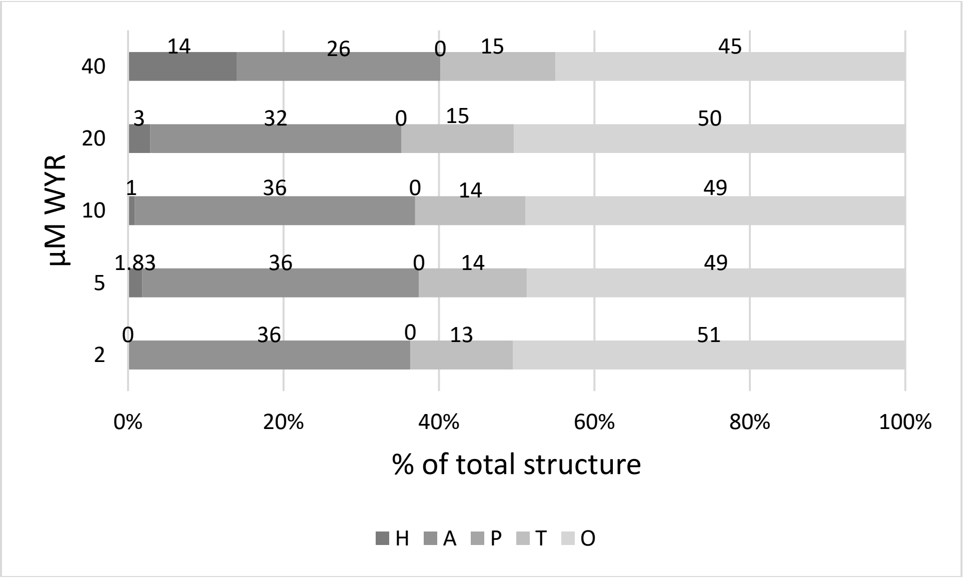

Supplementary Figure S2. WYR achieves higher helical content at increasing concentrations, as estimated by BeStSel (190-250 nm) (N=1).

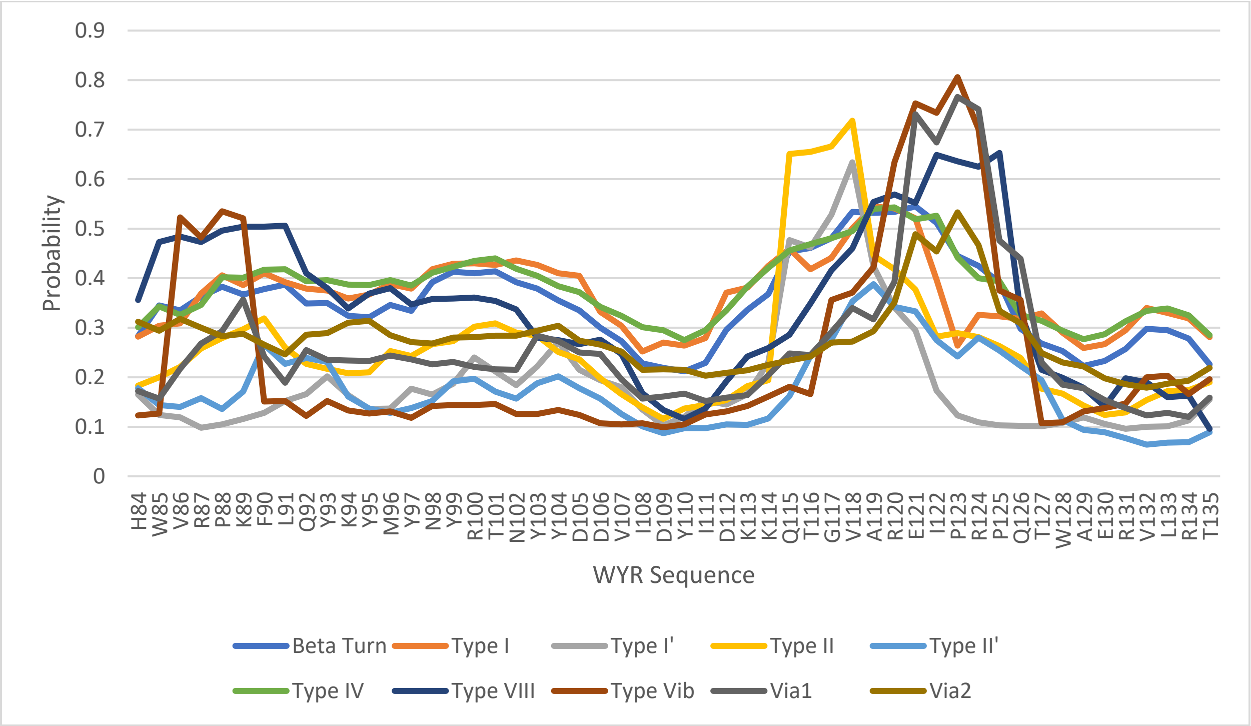

Supplementary Figure S3. NetTurnP prediction of beta turn propensity along the WYR sequence. The greatest propensity is predicted between K114 and Q126. The dark blue "Beta Turn" line represents probability of any type of beta turn.

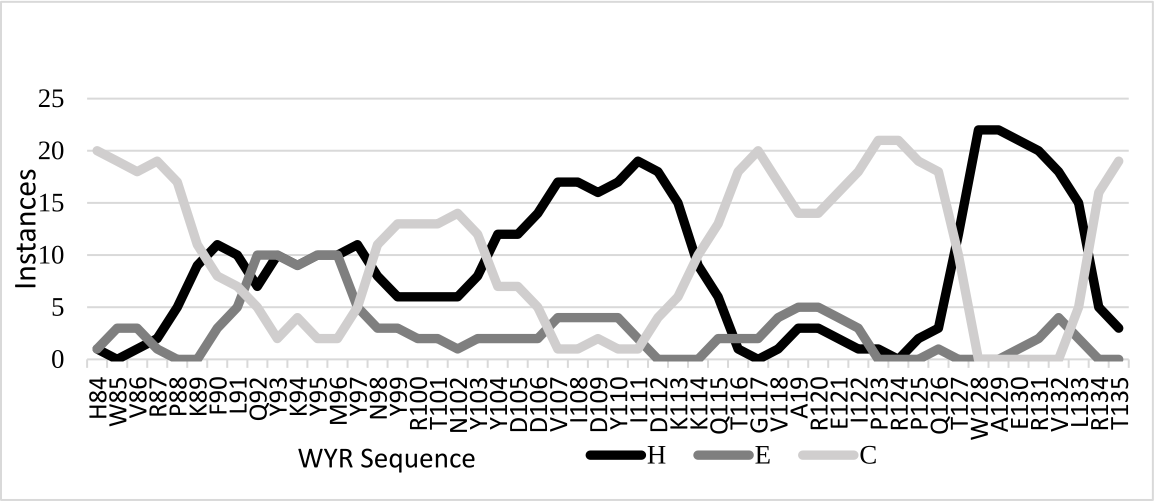

Supplementary Figure S4. Secondary structure designation of the WYR sequence for Helical (H), Extended beta strand (E), and Random Coil (C). Shown is the consensus of 22 programs (see main text for listing).

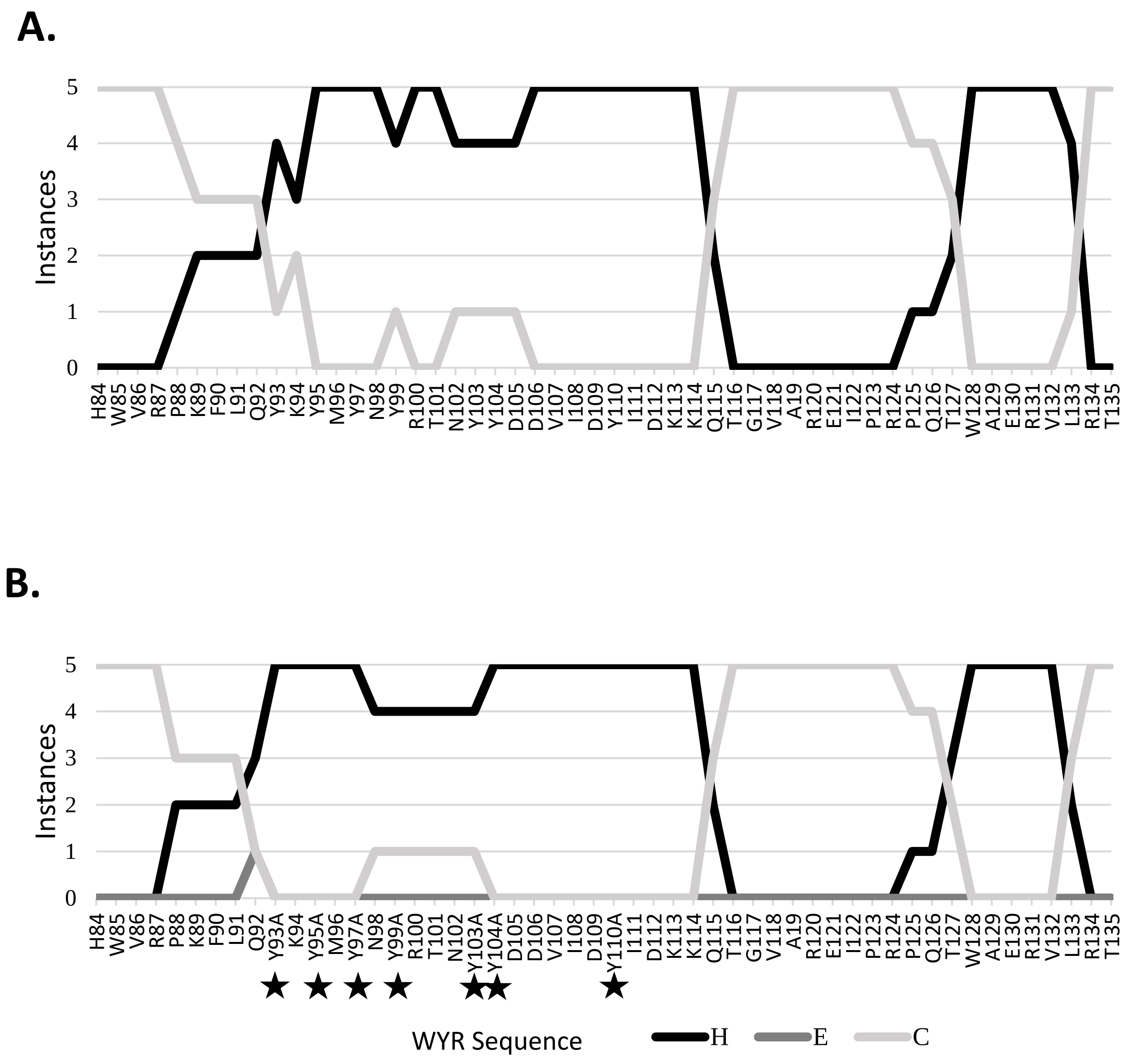

Supplementary Figure S5. (A) Secondary structure designation for WYR structure from 5 programs capable of finding Extended beta strand content (E) but only detecting Helical (H) or Random Coil (C) content. (B) Secondary structure designations for WYR from the same 5 programs with all tyrosines have been changed to alanines- shown on the x-axis with stars.

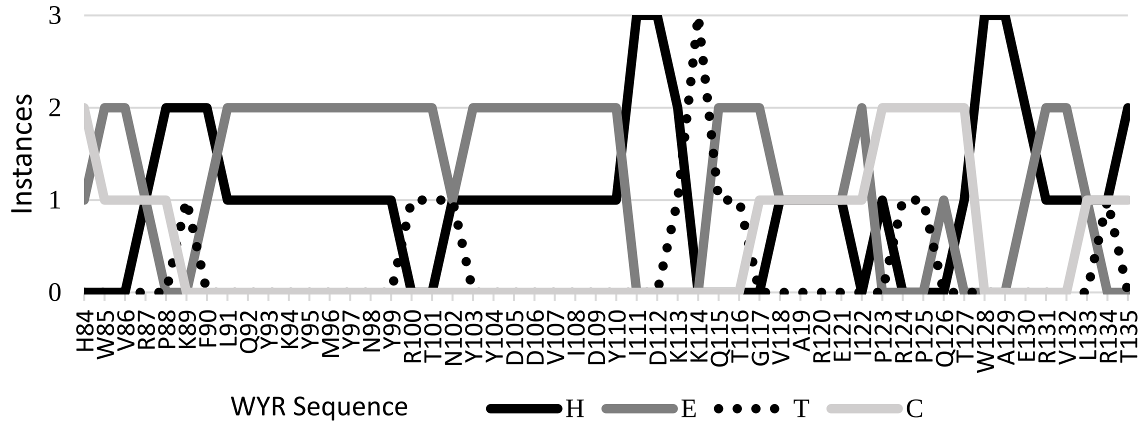

Supplementary Figure S6. Secondary structure designation from three turn-inclusive programs finding Helical (H), Extended beta (E), Turn (T), and Coil (C) content.

	92 102 112 122 132
WYR	HWVRPKFLQYKYMYNYRTNYYDDVIDYIDKKQTGVAREIPRPQTWAERVLRT
GOR1	eeeehtheeeeeeeeeeeteeeeeeeehhhteeeeeeeecccccchheeeeth

Supplementary Figure S7. Secondary structure designation by GOR1. ‘e’ is for extended beta strand, ‘h’ is for helical, ‘t’ is for turn and ‘c’ is for random coil identification.

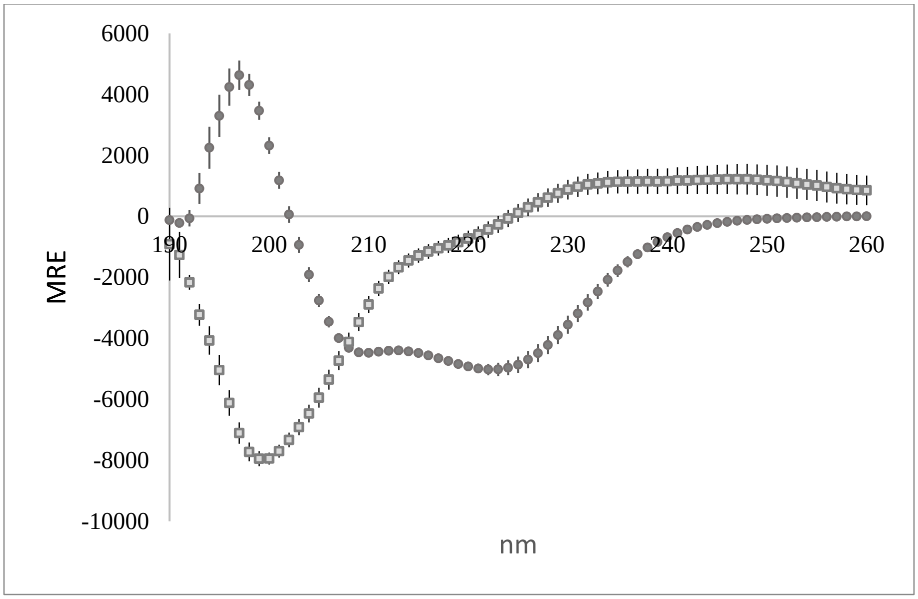

Supplementary Figure S8. WYR-alone (light grey squares) and LMM-alone (dark grey circles) Mean Residue Ellipticity (MRE) at 10 μM.

Supplementary Table S1. BeStSel structural predictions for Act(sep), Act(comb), Theo(sep) and Theo(comb) for 190-250 nm.

	**ACT(SEP)**	**THEO(SEP)**	**ACT(COMB)**	**THEO(COMB)**
**HELIX**	16.9%	21.4%	7.4%	16.5%
**ANTIPARALLEL**	17.9%	17%	31%	21.3%
**PARALLEL**	0%	2.1%	1.2%	0.4%
**TURN**	17.6%	13.3%	15.2%	15%
**OTHERS**	47.6%	46.1%	45.2%	46.8%

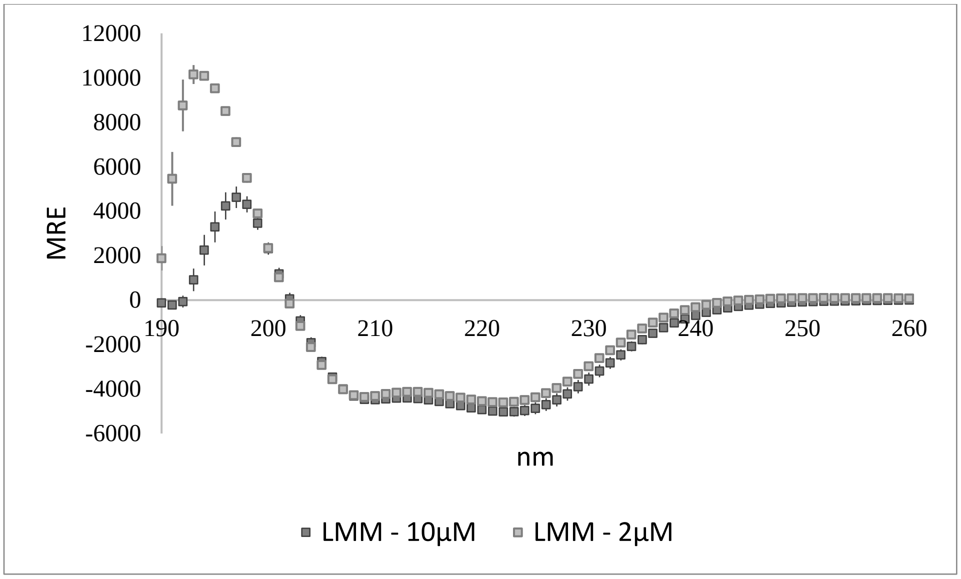

Supplementary Figure S9. MRE CD profile of LMM displays a characteristic helical profile at 10um and 2uM from 190-260 nm. The 193-194 nm maxima observed at 2 µM shows a bathochromic shift to 197-198 nm at the higher concentration.

Supplementary Table S2. Fractional alpha helical composition for 10 µM and 2 µM LMM spectral profiles according to various methods of calculation.

**Method**	**[LMM] 10 µM**	**[LMM] 2 µM**
208 nm magnitude	28.7%	28.5%
222 nm magnitude	19.1%	18.1%
230-240 nm slope	15.3%	14.2%
K2D	22%	19%
CONTIN (SMP180*)	8.2%	N/A
CDSSTR (SMP180*)	17.9%	8%
BeStSel (190-260)	15.6%	11.3%
222/208 ratio	1.17	1.08

The last row contains the average 222/208 ratio in which a ratio of 0.9-1 is indicative of alpha helical content and >1.1 is indicative of coiled-coil content. *The SP180 dataset was used for CONTIN & CDSSTR.

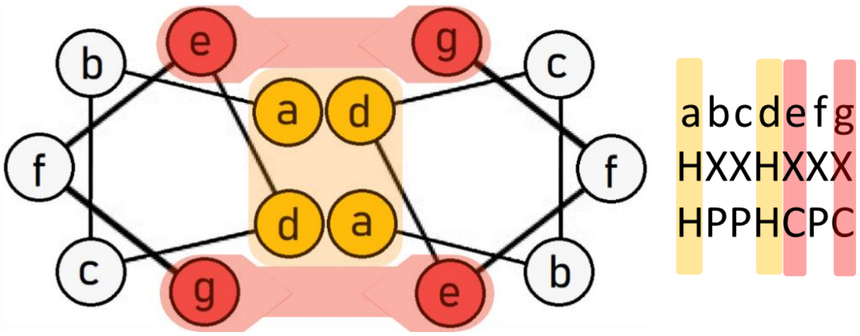

Supplementary Figure S10. Two alpha helices form a coiled-coil via their 'heptad' repeats in which positions 'a' and 'd' form a hydrophobic core and charged residues at positions 'e' and 'g' add further stabilization. Although HPPHCPC is the ideal heptad pattern, HXXHXXX is also an accepted heptad pattern. H=hydrophobic, P=polar, C=charged, X can be any type of residue.
